# Medical Students’ Confidence in Their Abilities and Barriers to Conducting Research: A Mixed-Methods Study

**DOI:** 10.7759/cureus.20896

**Published:** 2022-01-03

**Authors:** Robin J Jacobs, Joshua Caballero, Michael N Kane

**Affiliations:** 1 Medical and Behavioral Research, Health Informatics, Medical Education, Nova Southeastern University, Fort Lauderdale, USA; 2 Pharmacy, Larkin University, Miami, USA; 3 Social Work, Florida Atlantic University, Boca Raton, USA

**Keywords:** mixed methods, focus groups, barriers, medical education, preclinical, confidence, research, medical student

## Abstract

Background

Medical students’ confidence in their ability to conduct research has been speculated to be a significant factor influencing the engagement of research, yet their confidence may remain low. Moreover, it is unclear what barriers exist to high engagement in research before graduation. Hence, the objective of this study was to investigate medical students’ attitudes, confidence, and perceived barriers regarding conducting research.

Methodology

A cross-sectional study was conducted using quantitative and qualitative methods to investigate medical students’ attitudes, confidence, and perceived barriers regarding doing research. Quantitative data were collected from 141 first-year medical students via an online questionnaire in September 2020 asking about their perceptions toward participating in research while in school. Linear regression was performed to determine if certain perceptions and self-reported abilities would significantly contribute to research confidence. Additionally, focus groups were conducted with 30 students to assess student perceptions toward engaging in research. For qualitative data, an inductive thematic analysis was performed to allow for the patterns, themes, and categories to emerge.

Results

Regression modeling successfully explained 71% of the variance in predicting research confidence [F(3,137) = 116.91, p < 0.01] with an R^2^ of 0.719 (adjusted R^2^ = 0.713). Feeling secure in one’s research knowledge, greater belief in the ability to conduct research, and feeling research was important for their future career significantly contributed to greater research confidence. Five themes related to student attitudes and perceived barriers to conducting research in medical school emerged from the qualitative interviews.

Conclusions

Findings from this study may help medical educators better understand which attitudes are prevalent among medical students that may influence their confidence and ultimately affect their engagement in research during preclinical training. Unblocking barriers to research engagement and incorporating curricular strategies to help students gain practical experience and confidence in their research abilities may be warranted.

## Introduction

It has been widely understood that engaging in research activities during undergraduate medical training is associated with numerous research capabilities, including problem-solving skills, information retrieval skills, critical appraisal skills, and data collection skills [1-4]. Fostering the proliferation of new physician-scientists can help bridge the gap between practice and research, leading to advancements in medicine [5].

Medical students’ confidence in their ability to conduct research has been speculated to be a significant factor influencing the successful engagement of research. Given the demands and competing interests of formulating an undergraduate medical curriculum and results of attitudes of learners during medical training, it appears pivotal to investigate factors that promote student research during the undergraduate years.

The potential benefits for medical practice are significant as trainees develop knowledge and comfort with primary research resources as well as clinical reasoning, critical thinking, and problem-solving skills. Although some medical schools integrate research methods training and experiences into the curriculum, most are voluntary and offered as an elective course or extracurricular activities [6,7]. For novice researchers, a requirement to engage in research, including simple tasks such as accessing library resources for research, at the medical school level can produce anxiety, possibly adding to already tenuous confidence in their ability to master the medical curriculum [8-10].

Aside from lack of confidence, several barriers to learning and conducting research at the preclinical level have been reported, such as heavy demands and competing interests present for medical students, particularly in the first two years of training [11,12]. Hence, the objective of this study was to investigate preclinical medical students’ attitudes and self-assessment of their abilities and confidence along with perceived barriers regarding conducting research before taking the foundations of research course, supplemental research lectures, and a one-year research practicum mandatory at the medical school from which data for this study were collected. Based on the empirical literature and anecdotal evidence, this study sought to answer the following questions: (1) what are preclinical medical students’ experiences and attitudes towards learning about and conducting research? and (2) what factors significantly contribute to research confidence in this group?

## Materials and methods

Overview

This study aimed to investigate the attitudes and perceptions that significantly contribute to research confidence in preclinical medical students, including identifying barriers to conducting research while in medical school. Data were collected using an online quantitative questionnaire from preclinical medical students in a college of osteopathic medicine in Florida from September to October 2020. A linear regression analysis was conducted to investigate factors that may serve as significant contributors to research confidence and perceived barriers to conducting research among preclinical medical students. Qualitative focus groups were conducted in October 2020. Data from the quantitative questionnaire and qualitative focus groups were analyzed. The overarching premise is that the integration of the two approaches would provide an added benefit regarding the research objectives that a single approach may not sufficiently provide [13].

Participants

This study was approved by the Nova Southeastern University Institutional Review Board. To participate in the study, the participant must have been a preclinical student enrolled in the osteopathic medical program at the first author’s university. The survey was distributed online via an e-mail invitation using the institution’s medical student listservs. Participants were informed about the study via a cover letter that accompanied the survey. The survey took approximately 10-15 minutes to complete. Reminder notifications were sent at certain intervals to promote respondent participation and questionnaire completion and to thereby reduce non-response rates. In the cover letter, students were invited to participate in one of three one-time focus groups. Those students interested in participating in a focus group were asked to send their e-mail and their interest in participation to the researcher. Subsequently, the students were contacted for scheduling time for the groups. Participants were then scheduled on a first-come, first-serve basis. One group was scheduled and completed before starting the next group to allow for data saturation, in which case the third group would have been dropped (or a fourth one added). At the beginning of each Zoom focus group, participants received a consent letter and, after reading it and having questions answered by the researcher (if needed), verbally consented. The focus group sessions were designed to last up to an hour. It is unknown if the students who participated in the focus groups completed the anonymous quantitative questionnaire portion of the study. At the end of the focus group, each participant received an Amazon gift card in appreciation of their time.

Materials

Pertinent published studies, validated scales, and anecdotal evidence guided the creation of the 72-item Osteopathic Medical Student Research Questionnaire (OMS-RQ) to assess student attitudes, perceptions, and experiences with research. The following content areas were assessed: (1) attitudes toward research, including confidence; (2) perceived ability to conduct research tasks; (3) security in how well they understood research; (4) prior experience with research; (5) interest in research during medical school; and (6) future expectations. Semi-structured interview guides for the focus group interviews were developed by the researchers and included questions about students’ perceptions of their research ability and confidence, including barriers and facilitators to conducting research. The interview guide included the following items: tell us about your experiences with research activities (Probe: if you do not have any, what kind of experiences would you like to have?); what makes engaging in research interesting or not interesting for you? (Probe: what would be needed to make it interesting for you?); What are some of the reasons you have not/would not engage in research activities while in medical school? (Probe 1: can you describe elements of research you prefer?, probe 2: what barriers or facilitators do you perceive exist to conduct research while in medical school?); how do you perceive your abilities to conduct research?; and what are your thoughts about conducting research after graduation?

Quantitative assessment

The OMS-RQ was distributed during September and October 2020 to incoming first-year students via REDCap (http://projectredcap.org/), a user-friendly secure web application for building and managing online surveys and databases [14]. It contained Likert-format items, categorical items in which set choices were provided, and dichotomous-format items (yes/no). The questionnaire contained selected demographic data (e.g., age, sex, racial/ethnic identity). Validated scales regarding students’ attitudes (i.e., research confidence, self-perceived ability to perform research tasks) were included, all of which demonstrated good reliability. Also included was a measure to ascertain participants’ belief in how well they understood research.

Sample Characteristics

Single items were used to assess participants’ personal characteristics (e.g., age, sex, education).

Perceived Ability to Conduct Research

Students reported on a scale of 1-5 (1 = strongly agree, 5 = strongly disagree) how secure they felt in their understanding of certain research concepts (e.g., basic statistics, evidence-based medicine). Higher scores indicated greater levels of security in understanding research.

Security in One’s Knowledge of Research

To measure students’ security in their knowledge of research, five items were used (adapted from the Student Research Education & Opportunities Survey) [15]. The five-point Likert response set ranged from strongly agree to strongly disagree, with lower scores indicating higher security in understanding research. Items in this scale included the following: I feel secure in my understanding of medical articles; I feel secure in my understanding of basic statistics; I feel secure in my understanding of how to conduct a research study; I feel secure in my understanding of evidence-based medicine; and I feel secure in my understanding of research ethics.

Research Confidence

To measure research confidence, a scale measuring students’ self-reported confidence to complete 13 specific research tasks was used [16]. Items in this scale included increased confidence in the following areas: research participation; writing a literature review; performing a power and sample size analysis; summarizing and presenting project results; constructing a dataset; choosing statistical analyses; describing and summarizing the meaning of the results; and presenting the findings. The scale ranged from 1 to 10, whereby 1 indicated “cannot do at all” and 10 indicated “highly certain can do.” Higher scores indicated greater levels of confidence in conducting specific research tasks.

Importance of Research for Future Career

One item, “In your opinion, how important is research for your future career?,” was included in the survey. It was scored on a five-point scale (very important, important, moderately important, slightly important, not important).

Qualitative interviews via focus groups

Three focus groups were conducted using Zoom meeting software with preclinical medical students in October 2020. A semi-structured interview guide was created using pertinent published studies, anecdotal evidence, and findings from previous studies with this population asking participants about their research activities, interests, engagement (preferences and barriers), and abilities.

Data analysis

Quantitative Data Analysis

All the data were cross-checked for errors (e.g., out-of-range values, missing data, outliers). The distribution and dispersion of data were conducted using descriptive numerical summaries and graphical tools to assess assumptions and relationships among important variables. The Statistical Package for the Social Sciences version 27 (IBM Corp., Armonk, NY) was used to analyze the data from this study after extraction from REDCap [14]. All data were cross-checked for errors (e.g., out-of-range values, missing data, outliers). Surveys with more than one-third (33%) of missing data were considered incomplete and excluded from the data analysis. Internal consistency (Cronbach’s alpha) for scales was computed for the sample and compared with estimates from previous studies (if available) in which the instruments were used. For this study sample, internal consistency estimates for measures within the survey were favorable: measure of self-rated ability to conduct research (α = 0.91); security in one’s knowledge to conduct research (α = 0.79); and research confidence (α = 0.94).

Sample characteristics were summarized as frequency and percentage for discrete variables and as means and standard deviation for continuous variables. To determine which major study variables to use, a Pearson’s correlation coefficient (r) analysis was conducted before multiple regression. Variables that were statistically significantly correlated were entered into the multivariate linear regression analysis to identify the factors that significantly contributed to research confidence.

Pearson correlation coefficients (r) and multiple regression analysis were performed for hypothesis testing. A linear regression analysis was used for hypothesis testing to explore the contributions of preclinical medical students’ self-rated ability to conduct research, security in one’s knowledge to conduct research, and feeling that research was important for their future career on research confidence.

Qualitative Data Analysis

The focus group sessions lasted from 45 to 60 minutes. Data saturation was reached at the third focus group with a total sample size of 30 participants. Using an iterative process, qualitative data from the focus group interviews were hand-coded by the researchers, compared, and organized into themes to elucidate the findings from the quantitative survey. An inductive thematic analysis (ITA) was conducted to allow for the patterns, themes, and categories of analysis to emerge and to reveal data about participants’ views, opinions, knowledge, experiences, or values that may not be captured in quantitative assessments. Transcripts from the Zoom recordings combined with observational notes from the research assistants (observers during the focus groups) were used in an iterative process. The hand-coded data were organized by the researchers and research assistants into major themes that emerged. Themes and subthemes were cross-checked by the researchers for accuracy. Using an iterative process, the coded data were then organized into final major themes.

## Results

Out of the 402 students enrolled in the program, 141 first-year medical students completed the questionnaire (35% response rate). Overall, 81 (57.4%) identified as female and 60 (42.6%) identified as male. The mean age was 24.6 years (range = 21 to 43 years). In total, 20 (14.2%) earned a post-graduate degree (i.e., master’s degree) before attending medical school. Table 1 reports the summary statistics for the major study variables.

**Table 1:** Summary statistics of major study variables (N = 141). SD: standard deviation

	Minimum	Maximum	Mean	SD
Security in one’s research knowledge	1.00	4.25	2.3	0.7136
Perceived ability to conduct research	1.00	10.00	5.8	1.998
Research confidence	1.00	10.00	5.8	1.974
Importance of future research	1.00	5.00	2.4	1.146

Table 2 presents the correlations between the major study variables.

**Table 2:** Variables correlated with research confidence. *Correlation is significant at the 0.01 level (two-tailed).

	Research confidence
Security in one’s research knowledge	-0.658*
Perceived ability to conduct research	0.820*
Importance of research for future career	-0.316*

Table 3 shows the regression model that successfully explained 70% of the variance in predicting research confidence [F(3,137) = 116.91, p < 0.01] with an R^2^ of 0.719 (adjusted R^2^ = 0.713). Feeling secure in one’s knowledge about research, greater belief in the ability to conduct research, and feeling research was important in their future career significantly contributed to greater research confidence.

**Table 3:** Regression model for predicting research confidence. Predicted variable: research confidence. *Significant at a p-value of <0.01. SE: standard error

Constant	B	SE	Beta	t	P-value
	4.035	0.699		5.769	0.000*
Security in one’s research knowledge	-0.615	0.163	-0.222	-3.770	0.000*
Perceived ability to conduct research	0.640	0.059	0.647	10.821	0.000*
Importance of research for future career	-0.231	0.080	-0.134	-2.880	0.005*

Qualitative results

Many participants were interested in learning about and conducting research while in medical school, but most reported low confidence in doing so. However, if directed on how to do certain tasks, they would be able to accomplish them, and felt sure they could do it. Some participants were unclear what research entailed, having experience only through assisting faculty with ongoing lab work during their bachelor’s program. Some of the participants did not understand what research entails and were confused overall. Many participants reported that understanding research was essential to being a better physician.

The major themes that emerged from the interviews related to attitudes, knowledge, self-rated ability to conduct research, and barriers to conducting research in medical school include the following: (1) competing priorities, (2) low confidence in conducting research, (3) time constraints, (4) research not part of the curriculum, and (5) difficulty finding accessible research mentors with compatible interests.

Competing Priorities

Although many students felt the importance of research, they reported that their main concern and focus was on their medical curriculum coursework.

“I think it’s [research] super important, especially during school because like I feel like a lot of people don’t have research experience. Even if you don’t envision yourself having a career involved in research, I think it’s important to be aware of, kind of the basics of research.”

“Focusing on the school is the main priority.”

“You have to kind of weigh the benefits of becoming a doctor and putting the same time into studying or shuffling something to do some research.”

Low Confidence

Regarding barriers to conducting research while in medical school, many students reported a lack of confidence in their ability to get a project started due to a lack of experience with research.

“I think I would need someone to start me off doing it, and then I think I could theoretically plan a research project.”

“And like set up the methods … the organization of a research study that would be an issue for me is low because I don’t have much experience in that.”

Time Constraints

One of the largest barriers to conducting research was time.

“I would say that if it’s not made part of the curriculum, then the barrier is just for me will be just finding time.”

“In order to actually see anything happen, you have to put in so much time and you just have to keep going at it all the time or if not, nothing’s going to happen.”

Research Not a Part of the Curriculum

Curricular demands appeared to dovetail with time constraints.

“It’s not part of the curriculum and so adding more on top of that would be like pretty difficult.”

“If it’s [research] not made part of the curriculum, then the barrier is just for me will be just finding time … I’m having a hard enough time just figuring out how to do it.”

Difficulty Finding Accessible Research Mentors With Compatible Interests

“Especially with the number of students in our medical school, it’s tough to connect with professors individually, and being virtual it’s tough to meet with professors in person … and build a relationship with them.”

“Fields that interest you, you know, if you have something really specific in mind and you just can’t find the right person, or you don’t know how to start doing about finding that right person.”

“Having the skill set being taught to you in some way that you’ll be able to understand how to start a research project how to do a methods section, how to understand the nuts and bolts of what research is, and how to do a research project.”

“Finding, you know, the right professor or mentor … and research that is actually of interest to you that’s worth that extra time.”

## Discussion

This study identified several factors relevant to predicting research confidence and highlighted some of the barriers to conducting research.

Security

The mean score for this measure was 2.3 (on a scale of 1-4, whereby lower scores indicate higher levels of security in understanding research), indicating the overall sample was not completely secure in their knowledge of research. This finding was not surprising given the participants had no prior graduate-level training in research. The finding that feeling secure in one’s understating of research was associated with research confidence was not surprising. Logically, feeling secure in one’s knowledge and understanding of a subject would enhance the belief in one’s capacity to execute behaviors necessary to produce specific performance attainments. It may not be enough for students to possess the requisite knowledge and skills to perform research tasks; they also must have the conviction that they can successfully perform them [17-19]. This finding highlights the need for teaching research in a way that instills security in having the knowledge to carry out the tasks at hand.

Perceived ability

Although students expressed concerns about their ability to conduct research and faced challenges, their interest (as reported in the qualitative interviews) in strengthening their ability was high. The mean score for perceived ability to conduct research was 5.8 (on a scale of 1-10), indicating that many participants did not perceive their ability as optimum. Not having previous research experience, it is logical that students perceived their research abilities to be lacking. Higher scores on perceived ability to conduct research significantly contributed to research confidence. Qualitative responses from students indicated that they felt more experience with research will help them increase their ability to conduct autonomous research. Further, medical graduates are expected to be skilled in research, and gaining research experience early in their training may help them feel confident in their abilities.

Importance of research for a future career

Most students reported that research is important for their future career, and this idea significantly contributed to their research confidence. The mean score for this item, on a scale of 1-5, was 2.4, indicating that not all students felt that research was important. However, in the qualitative interviews, students maintained the perception that knowing how to conduct research can be useful, if not essential, in today’s competitive professional medical workforce. Many students were aware that research experience culminating in publications and presentations during medical school has become increasingly important in being selected for positions in residency training programs [20].

Research confidence

Confidence in conducting research can increase for medical students through exposure to research methods training, mentoring, and successful completion of research projects. The mean score for research confidence, whereby 1 signifies “cannot do at all” and 10 signifies “highly certain can do,” was 5.8, indicating that, on average, students felt some level of confidence, but many students still scored quite low. Research education and opportunities are an important part of undergraduate medical education. However, the integration of research training into undergraduate medical curricula is inconsistent, varying from elective research courses or activities to mandatory didactic classes [21-28]. There exists a lack of adequate and uniform educational experiences during medical school to help students develop confidence in their research ability, not just in their clinical skills [1,2].

Barriers to conducting research

Although not all participants stressed an interest in research, those who did report interest indicated there were barriers (confidence notwithstanding) that made it difficult for them to successfully engage in research projects. Several students commented that they would feel comfortable doing certain research-related tasks if there was someone to instruct and guide them along the way. Many of the barriers had more to do with logistics rather than confidence or motivation (e.g., competing priorities, time constraints, research seen as an extracurricular activity one must complete on one’s own time, lack of accessible faculty mentors). These findings are supported by previous studies [3,11,12,28].

Limitations

First, this study used a cross-sectional survey design and generalizations cannot be made regarding changes or trends over time, the directionality of influence, or cause-and-effect relationships. Second, multi-site data collection might have provided a more diverse sample of respondents, which limits the ability to generalize findings to all preclinical medical students. Third, there were several disadvantages of conducting interviews and research via surveys over the Internet, such as limited respondent availability or willingness to respond without a person in front of them. Lastly, challenges of collecting research with medical students may have been exacerbated during the height of the coronavirus disease 2019 pandemic when the data for this study were collected. The pandemic caused a substantial increase in research activity while restricting data collection methods, leading to a surge in online survey studies, which may have contributed to survey fatigue.

## Conclusions

Results from the analyses indicated that several factors (i.e., security in one’s knowledge about research, perceived ability to conduct research, and importance of research for future career) significantly contributed to research confidence in first-year medical students. The study findings also revealed some of the barriers that hinder students from conducting research during their undergraduate medical training, such as competing priorities, lack of confidence in conducting research, time constraints, research not infused in the curriculum, and inability to find accessible mentors with compatible interests.

Findings from this study may help medical educators better understand which attitudes and beliefs are prevalent among medical students that may influence their confidence and ultimately affect their engagement in research while in school. Incorporating innovative curricular strategies to help students understand the relevance of research and how it impacts daily medical practice may be warranted. Moreover, research skills are becoming increasingly important for medical students competing for residency placements. To develop the next generation of physicians who will think critically, practice evidence-based medicine, participate in meaningful and ethical research, and pose thoughtful questions as life-long learners to enhance patient care, more thought needs to be given to how best to engage students in research early during the preclinical phase of medical training.

## References

[1] Houlden RL, Raja JB, Collier CP, Clark AF, Waugh JM (2004). Medical students' perceptions of an undergraduate research elective. Med Teach.

[2] Ogunyemi D, Bazargan M, Norris K (2005). The development of a mandatory medical thesis in an urban medical school. Teach Learn Med.

[3] Frishman WH (2001). Student research projects and theses: should they be a requirement for medical school graduation?. Heart Dis.

[4] Solomon SS, Tom SC, Pichert J, Wasserman D, Powers AC (2003). Impact of medical student research in the development of physician-scientists. J Investig Med.

[5] Ommering BW, van Diepen M, van Blankenstein FM, de Jong PG, Dekker FW (2020). Twelve tips to offer a short authentic and experiential individual research opportunity to a large group of undergraduate students. Med Teach.

[6] Chang Y, Ramnanan CJ (2015). A review of literature on medical students and scholarly research: experiences, attitudes, and outcomes. Acad Med.

[7] Zier K, Friedman E, Smith L (2006). Supportive programs increase medical students' research interest and productivity. J Investig Med.

[8] Guthrie EA, Black D, Shaw CM, Hamilton J, Creed FH, Tomenson B (1995). Embarking upon a medical career: psychological morbidity in first year medical students. Med Educ.

[9] Sajjad A, Muhammad I, Khan A (2021). Prevalence of library anxiety among undergraduate medical students. Libr Philos Pract.

[10] Quek TT, Tam WW, Tran BX, Zhang M, Zhang Z, Ho CS, Ho RC (2019). The global prevalence of anxiety among medical students: a meta-analysis. Int J Environ Res Public Health.

[11] Kumar J, Memon A, Kumar A, Kumari R, Kumar B, Fareed S (2019). Barriers experienced by medical students in conducting research at undergraduate level. Cureus.

[12] Siemens DR, Punnen S, Wong J, Kanji N (2010). A survey on the attitudes towards research in medical school. BMC Med Educ.

[13] Teddlie CB, Tashakkori AM (2009). Foundations of mixed methods research: integrating quantitative and qualitative approaches in the social and behavioral sciences.

[14] Harris PA, Taylor R, Thielke R, Payne J, Gonzalez N, Conde JG (2009). Research electronic data capture (REDCap)--a metadata-driven methodology and workflow process for providing translational research informatics support. J Biomed Inform.

[15] Klowak J, Elsharawi R, Whyte R, Costa A, Riva J (2018). Predictors of medical student interest and confidence in research during medical school. Can Med Educ J.

[16] DeVoe P, Hess M (2018). Can research participation positively impact medical student research self-efficacy?. MedEdPublish.

[17] Bandura A (1977). Self-efficacy: toward a unifying theory of behavioral change. Psychol Rev.

[18] Bandura A (1986). Social foundations of thought and action: a social cognitive theory. https://psycnet.apa.org/record/1985-98423-000.

[19] Bandura A (1997). Self-efficacy: the exercise of control. https://psycnet.apa.org/record/1997-08589-000.

[20] Collier AC (2012). Medical school hotline: importance of research in medical education. Hawaii J Med Public Health.

[21] Burgoyne LN, O'Flynn S, Boylan GB (2010). Undergraduate medical research: the student perspective. Med Educ Online.

[22] Murdoch-Eaton D, Drewery S, Elton S (2010). What do medical students understand by research and research skills? Identifying research opportunities within undergraduate projects. Med Teach.

[23] van Eyk HJ, Hooiveld MH, Van Leeuwen TN, Van der Wurff BL, De Craen AJ, Dekker FW (2010). Scientific output of Dutch medical students. Med Teach.

[24] Laskowitz DT, Drucker RP, Parsonnet J, Cross PC, Gesundheit N (2010). Engaging students in dedicated research and scholarship during medical school: the long-term experiences at Duke and Stanford. Acad Med.

[25] O'Connor Grochowski C, Halperin EC, Buckley EG (2007). A curricular model for the training of physician scientists: the evolution of the Duke University School of Medicine curriculum. Acad Med.

[26] Salam A, Hamzah JC, Chin TG, Siraj HH, Idrus R, Mohamad N, Raymond AA (2015). Undergraduate medical education research in Malaysia: time for a change. Pak J Med Sci.

[27] Patel S, Walsh CM, Udell JA (2019). Exploring medically-related Canadian summer student research programs: a National Cross-sectional Survey Study. BMC Med Educ.

[28] Oliveira CC, de Souza RC, Abe EH, Silva Móz LE, de Carvalho LR, Domingues MA (2014). Undergraduate research in medical education: a descriptive study of students' views. BMC Med Educ.

